# Clinical evidence for orphan medicinal products-a cause for concern?

**DOI:** 10.1186/1750-1172-8-164

**Published:** 2013-10-16

**Authors:** Eline Picavet, David Cassiman, Carla E Hollak, Johan A Maertens, Steven Simoens

**Affiliations:** 1 KU Leuven, Department of Pharmaceutical and Pharmacological Sciences, Herestraat 49, PO box 521, Leuven 3000, Belgium; 2 Department of Hepatology, University Hospital Leuven, Herestraat 49, Leuven 3000, Belgium; 3 Department of Internal Medicine, Division of Endocrinology and Metabolism, Academic Medical Center, Meibergdreef 9, Amsterdam, AZ, 1105, The Netherlands; 4 Department of Hematology, University Hospital Leuven, Herestraat 49, Leuven 3000, Belgium

**Keywords:** Quality assessment, Quality tool, Pivotal study

## Abstract

**Background:**

The difficulties associated with organising clinical studies for orphan medicinal products (OMPs) are plentiful. Recent debate on the long-term effectiveness of some OMPs, led us to question whether the initial standards for clinical evidence for OMPs, set by the European Medicines Agency (EMA) at the time of marketing authorization, are too low. Therefore, the aim of this study was to quantitatively evaluate the characteristics and quality of clinical evidence that is presented for OMPs to obtain marketing authorization in Europe, using the new and validated COMPASS tool.

**Methods:**

We quantitatively assessed the characteristics and quality of clinical evidence of the pivotal studies of 64 OMPs as described in the European Public Assessment Report and/or the Scientific Discussion document prepared by the Committee for Human Medicinal Products of the EMA.

**Results:**

The 64 OMPs were altogether authorized for 78 orphan indications, for which 117 studies were identified as 'pivotal’ or 'main’ studies. In approximately two thirds of the studies, the allocation was randomized (64.8%) and a control arm was used (68.5%). Half of the studies applied some type of blinding. Only a minority (26.9%) of the studies included a Quality-of-Life (QoL) related endpoint, of which a third claim an improvement in QoL. Upon analyzing the quality of reporting, we found that some aspects (i.e. the endpoints, the sampling criteria, and the interventions) are well described, whereas other items (i.e. a description of the patients and of potential biases) are not reported for all studies.

**Conclusions:**

In conclusion, the pivotal studies that are the basis for marketing authorization of OMPs are a cause for concern, as they exhibit methodological flaws i.e. the lack of QoL-related endpoints as outcome, lack of blinding in the study design and the use of surrogate endpoints. Additionally, there are shortcomings in the reporting of those studies that complicate the interpretation. A more demanding regulatory process for OMPs is needed to guide evidence-based clinical decision-making.

## Background

The difficulties associated with organising clinical studies for orphan medicinal products (OMPs) are plentiful. Because of the small number of eligible patients, it can prove difficult to enroll a sufficient number of patients [1,2]. In such small studies, several problems can arise; firstly the validity of the results may be questionable. Secondly, there is a risk of not being able to demonstrate an effect in trials with complex patient populations which are more prone to variability and statistical challenges [3]. Additionally, rare diseases are frequently life-threatening and no treatments are available, giving rise to ethical issues related to assigning patients to a placebo treatment. Finally, the interpretation is complicated due to the heterogeneous and unpredictable presentation of the rare diseases and the use of surrogate endpoints, while demonstration of clinically relevant effectiveness may be only evident after many years [1,2].

In some cases, the use of an orphan medicinal product has been well established in clinical practice. For example, zinc has been used in the treatment of Wilson’s disease since 1958 and its effectiveness has been extensively documented [4]. In other cases, the level of clinical evidence is questionable. Joppi *et al*. expressed concerns about the general lack of efficacy data of orphan medicinal products [5]. Kesselheim *et al.* compared pivotal studies used to authorize non-orphan cancer drugs to those of orphan cancer drugs and found that the latter are more likely to be smaller, nonrandomized, unblinded and using surrogate end points [6]. Putzeist *et al.* found that for three out of four licensed OMPs approval was based on robust randomized studies and endpoints that were considered clinically relevant [7]. In contrast, a Belgian study argued that the clinical evidence presented at the time of reimbursement is poor for most orphan medicinal products [8].

At the moment, it is questionable whether timely access to new OMPs can be reconciled with quality of clinical evidence. This also leads to difficult reimbursement decisions as member states have different strategies for implementation of OMPs after central EU authorization. Indeed, local reimbursement agencies sometimes tend to attribute a special status to OMPs, in which reimbursement is granted, in spite of high prices and undemonstrated effectiveness. Other agencies ask for additional (cost-) effectiveness studies at a national level. Several authors have argued that the prioritization of rare diseases is not based on scientific evidence [8,9]. At the time of marketing authorization, the European Medicines Agency (EMA) can deal with incomplete clinical evidence by granting conditional marketing authorization or marketing authorization under exceptional circumstances. Recent debate on the long-term effectiveness of some OMPs, led us to wonder whether the initial standards for clinical evidence for OMPs, set by EMA at the time of marketing authorization, are too low. For example, the authors of a Cochrane review concluded that six poor quality controlled studies provide no evidence for the use of both agalsidase alfa and beta to treat Fabry disease [10]. Long term effectiveness of enzyme replacement therapy (ERT) for Fabry disease, in combination with supportive care, lowers the risks of developing complications, but does not prevent disease progression [11].

Therefore, the aim of this study was to quantitatively evaluate the characteristics and quality of clinical evidence that is presented for OMPs authorised in the EU up to 1^st^ July 2012 using the new and validated COMPASS tool [12].

## Methods

### COMPASS-Clinical evidence of orphan medicinal products-an assessment tool

The development and validation of the COMPASS tool is described in detail elsewhere [12]. Briefly, the tool consists of three parts and is to be completed based on information provided on the Orphanet website and in European Public Assessment Report (EPAR) and/or the Scientific Discussion (SD) document prepared by the Committee for Human Medicinal Products (CHMP) of the EMA. The first part collects general descriptive information about the OMP and its marketing authorization. The second part focuses on the assessment of the methodological quality (i.e. specifically related to study design, patient and study population, control arm, blinding, randomization and allocation, outcomes, adherence and statistical analysis) of the pivotal clinical study. The last part assesses quality of reporting as shortcomings in the reporting can complicate the interpretation of the methodological quality. The tool itself does not attempt to score or rank the quality of clinical evidence, but rather to give an outline of various, key elements with respect to quality of clinical evidence.

### Data source

We included all OMPs (n = 64) that were listed as authorized on the website of the EMA on July 1^st^ 2012 (i.e. centrally approved OMPs) [13]. The same data sources, as used during the development of the tool, were consulted [12]. Again, the study was restricted to studies that were described as 'pivotal’ or 'main’ clinical studies. The analyses were performed per study, as opposed to per orphan medicinal product, due to possible methodological differences between the studies. For practical and privacy reasons, we did not have access to the original documents submitted to EMA. However, we anticipated that the publicly EPARs sufficiently reflect these original documents. Additionally, we did not consult publications due to unsystematic reporting and publication bias. The power of a study was defined as the probability of reaching a true positive conclusion [14].

One rater (E.P.) completed the template for all OMPs (n = 64), whereas another rater (S.S.) analysed a random sample of OMPs (n = 29). The raters completed the tool independently and once-only. Additionally, raters were blinded with respect to results of others. The same information was available to all raters. All disagreements between two raters were resolved upon consensus. Additionally, a third rater (D.C.) (i.e. a physician) specifically answered the question “Is the duration of the trial relevant to the natural history of the disease?” for all OMPs. Upon disagreement between the raters, the assessment of D.C. was considered decisive. After data collection, E.P. was responsible for comparison of the results. With a view to increasing content validity, two physicians with an expertise in metabolic disorders (C.H.) and hematology (J.M.) examined and commented on the results obtained for respectively three enzyme replacement therapies (agalsidase alfa, agalsidase beta, laronidase) and three hematological medicines (ofatumumab, clofarabine, cladribine).

### Analysis

All analyses were performed using MS Office Excel 2010.

## Results

### Analysis of the indications

We included 64 OMPs in the analysis of which 46 received a normal marketing authorization, three obtained conditional marketing authorization and 15 were authorized under exceptional circumstances. The OMPs were altogether authorized for 78 orphan indications (54 for one indication, nine for two indications and one for six indications). Estimates of prevalence of each rare disease in which an indication is authorized were pulled from Orphanet (n = 56) or the EPAR (n = 9). No estimates of prevalence were found for 13 rare diseases. The sponsor requested protocol assistance for just one in four of the indications as shown in Table 1.

**Table 1:** Characteristics of the indications (n = 78)

	**Number of indications (%)**
**Prevalence of the rare disease in which the indication is authorized**	
Between 4/10 000 and 5/10 000	1 (1.3%)
Between 3/10 000 and 4/10 000	3 (3.8%)
Between 2/10 000 and 3/10 000	5 (6.4%)
Between 1/10 000 and 2/10 000	14 (17.9%)
Between 1/10 000 and 2/10 000	42 (53.8%)
**Requested protocol assistance**	
Yes	22 (28.2%)
No	15 (19.2%)
Not reported	41 (52.6%)
**Performed dose finding study**	
Yes	44 (56.4%)
No	25 (32.0%)
Not reported	9 (11.5%)

### Analysis of the characteristics of the pivotal studies

For these 78 indications, 117 studies were identified as 'pivotal’ or 'main’ studies. For 45 indications, one pivotal study was reported; for 23 indications there were two pivotal studies; for three indications there were three pivotal studies and for two indications there were four pivotal studies. Additionally, for six indications, reports from the literature were added as pivotal evidence. For three indications, the pivotal evidence consisted only of literature reports. No pivotal efficacy study was submitted for 6-mercaptopurine monohydrate as treatment for acute lymphoblastic leukemia. Due to the conciseness and lack of information in the EPAR, nine pivotal studies were excluded from the analysis i.e. eight that consisted of literature reports and one based on patient registries.

The characteristics of the remaining 108 pivotal studies (for 59 OMPs) are summarized in Table 2. As a primary endpoint, less than one in five studies used at least one hard endpoint. Hard endpoints represent definitive outcomes of the disease process (i.e. overall survival). Secondary endpoints were also mostly surrogate endpoints (i.e. intermediary endpoints such as biomarkers). For nine pivotal studies the secondary endpoint was not defined or not reported. The size of the study population ranged between 7 and 976 individuals, with a median of 113 (IQR 222).

**Table 2:** Characteristics of the pivotal studies (n = 108)

	**Number of pivotal studies (%)**
**Study phase**	
Phase III	50 (46.3%)
Phase II-III	9 (8.3%)
Phase II	39 (36.1%)
Phase I	2 (1.9%)
Not reported/Not applicable	8 (7.4%)
**Center characteristics**	
Multicenter	91 (84.3%)
Mono-center	5 (4.6%)
Not reported	12 (11.1%)
**International character of the study**	
Multinational	15 (13.9%)
Mono-national	65 (60.2%)
Not reported	28 (25.9%)
**Primary endpoint**	
At least one hard endpoint	21 (19.4%)

Table 3 shows that most of the pivotal studies were made available through publication in peer-reviewed international journals. Similarly, a large majority of the studies was approved by an ethics committee (in accordance with Good Clinical Practice guidelines). More than a quarter of the studies were not registered at ClinicalTrials.gov or EUdraCT.

**Table 3:** Publication and registration of the pivotal studies (n = 108)

	**Number of pivotal studies (%)**
**Publication in peer-reviewed international journal**	
Yes	76 (70.3%)
**Registration on EUdraCT**	
Yes	21 (19.4%)
**Registration on ClinicalTrials.gov**	
Yes	52 (48.1%)
**Approval by Ethics Committee**	
Yes	77 (71.3%)
No	2 (1.9%)
Not reported	29 (26.9%)

### Analysis of the methodological quality of the pivotal studies

A first aspect related to the methodological quality of the studies is the choice of study design. Ethical considerations (such as, that it was deemed unethical to use blinding or a placebo) were reported to have influenced the choice of study design for 14 pivotal studies. Other practical considerations (such as, the complexity of blinding) influenced the choice of study design for 31 pivotal studies. In most cases the allocation was randomized and a control arm was used in the study design. For a small minority of the studies, we were able to verify that there was similarity between treatment and placebo groups at baseline. Also, just half of the studies applied some type of blinding (Table 4).

**Table 4:** Study design of the pivotal studies (n = 108)

	**Number of pivotal studies (%)**
**Control arm**	
No control	34 (31.5%)
Controlled	74 (68.5%)
Historical control	2 (1.9%)
Different dosages of the OMP	11 (10.2%)
Placebo	49 (45.4%)
Active comparator (or standard of care)	17 (15.7%)
Similarity at baseline	
Yes, statistically verified	13 (12.0%)
Likely, but not statistically verifiable	41 (38.0%)
Not likely, but not statistically verifiable	4 (3.7%)
No, statistically verified	1 (0.9%)
Not reported	15 (13.9%)
**Randomized allocation**	
No	38 (35.2%)
Yes	70 (64.8%)
Valid method of randomization	25 (23.1%)
Invalid method of randomization	2 (1.9%)
Not reported	43 (39.8%)
**Blinding**	
No (open-label)	44 (40.7%)
No, but justified	10 (9.3%)
Yes	54 (50.0%)
Blinding of the care provider	53 (49.1%)
Blinding of the outcomes assessor	12 (11.1%)
Blinding of the patient	54 (50.0%)

For the majority of the orphan medicinal products there is a least one randomized study and at least one study where a placebo or the standard of care treatment was used in the control arm. For just over half of the OMPs at least one blinded study was performed (Table 5).

**Table 5:** Summary of study design of all orphan medicinal products (n = 59)

	**Number of OMPs (%) (n = 59)**
**Control arm**	
At least one study with placebo or standard of care as control	44 (74.6%)
**Randomization**	
At least one study with random design	46 (78.0%)
**Blinding**	
At least one study with double blind design	34 (57.6%)

Secondly, we analysed whether the study population adequately represents, or in other words, reflects the possible heterogeneity of the entire patient population (Table 6). For example, in one case the study population was considered inappropriate because only patients older than seven years were included, to study a disease that first occurs in infants (i.e. galsulfase study ASB-03-05). However, due to lack of patient demographics and/or the inclusion and exclusion criteria this query could not be answered for 45 studies. For almost half of the studies, it was not reported whether a priori power calculations were made (Table 6).

**Table 6:** Characteristics of the study population in the pivotal studies (n = 108)

	**Number of pivotal studies (%)**
**Study population represents the patient population?**	
Yes	53 (49.1%)
No	11 (10.2%)
Don’t know	44 (40.7%)
**A priori power calculations**	
Yes, and the required number of inclusions was achieved	51 (47.2%)
Yes, but the required number of inclusions was not achieved	7 (6.5%)
Yes, but it is unclear if the required number of inclusions was achieved	3 (2.8%)
No / Not reported	47 (43.5%)

Only a minority (26.9%) of studies included a Quality-of-Life (QoL) related endpoint. Additionally, an improvement in QoL was claimed in just a third of those (Table 7). The duration of all studies was deemed relevant, i.e. sufficient to show meaningful clinical benefits, with respect to the disease for fewer than 80% of the studies. The few studies that measured patient adherence, all report high adherence. As a final item related to methodological quality, we performed a descriptive examination of the statistical analysis of the pivotal studies (Table 8). The appropriateness of the analysis plan was assessed based on the information provided in the EPAR. The following aspects of the analysis plan were evaluated: statistical hypothesis, statistical testing, missing values, significance level, outliers, sensitivity analysis, and outcomes (i.e. p-values, confidence intervals). The statistical analysis plan was deemed inappropriate in some, not predefined, circumstances. For example, if there was inappropriate use of the last observation carried forward (LOCF) method to replace missing values. Finally, summary statistics (at baseline and at outcome) with the appropriate probability values were not always provided.

**Table 7:** Methodological conduct of the pivotal studies (n = 108)

	**Number of pivotal studies (%)**
**QoL-related endpoint**	
No / Not reported	79 (73.1%)
Yes, using a disease-specific QoL scale	9 (8.3%)
Yes, using a generic QoL scale	16 (14.8%)
Yes, using both a disease-specific and a generic QoL scale	4 (3.7%)
**Improvement in QoL (n = 29)**	
Yes	10 (34.5%)
No	14 (13.0%)
Not reported	5 (4.6%)
**Relevant duration of the study**	
Yes	86 (79.6%)
**Non-adherence to the study protocol**	
Yes, by certain subjects	7 (6.5%)
Yes, by the researcher(s)	15 (13.9%)
Yes, by both	7 (6.5%)
No / Not reported	79 (73.1%)
**Assessment of patient adherence**	
Yes	17 (15.7%)
No / Not reported	91 (84.3%)

**Table 8:** Statistical analysis of the pivotal studies (n = 108)

	**Number of pivotal studies (%)**
**Appropriate statistical analysis**	
Yes	48 (44.4%)
No	41 (38.0%)
Don’t know (too few details)	19 (17.6%)
**Summary statistics at *baseline***	
Yes	71 (65.7%)
With statistical testing	7 (6.5%)
**Summary statistics at *outcome***	
No	5 (4.6%)
Yes, for all endpoints	44 (40.7%)
Statistically tested	14 (13.0%)
Not statistically tested	30 (27.8%)
Yes, partially	45 (41.7%)
Statistically tested	31 (28.7%)
Not statistically tested	14 (13.0%)
Yes, only primary	14 (13.0%)
Statistically tested	10 (9.3%)
Not statistically tested	4 (3.7%)
**Intention-to-treat analysis**	
Yes	74 (68.5%)
No	8 (7.4%)
Not applicable / Not reported	26 (24.0%)
**Reported with respect to patients lost to follow-up**	
Reasons for loss to follow-up	70 (64.8%)
Characteristics	2 (1.9%)
Not applicable	8 (7.4%)

### Analysis of the quality of reporting of the pivotal studies in the EPAR

Finally, we examined the quality of reporting, as carried out by the Committee for Human Medicinal Products (CHMP) in the EPAR, for 108 pivotal studies. We found that some aspects (i.e. the endpoints, the sampling criteria, and the interventions) are well described for the majority of the studies (Table 9). However, other items such as a description of the patients and of potential biases and confounders are not reported for all studies. Additionally, the point estimates and the measures of variability for all endpoints were reported for only 15 studies. For two studies, neither the point estimates nor the measures of variability were reported.

**Table 9:** Quality of reporting of the pivotal studies (n = 108)

	**Number of pivotal studies (%)**
**Reporting of an a priori stated research hypothesis**	
Yes	71 (65.7%)
No	12 (11.1%)
Not reported	25 (23.1%)
**Reporting of main endpoints**	
Yes	101 (93.5%)
**Reporting of sampling criteria**	
Yes	100 (92.6%)
**Description (i.e. age, gender) of patients included**	
Yes	70 (64.8%)
**Reported possible bias and/or confounding**	
Yes	45 (41.7%)
**Reporting of the intervention**	
Yes	107 (99.1%)
**Reporting of point estimates**	
Yes, for all primary and secondary endpoints	39 (36.1%)
Yes, but partially	67 (62.0%)
No	2 (1.9%)
**Reporting of measures of variability**	
Yes, for all primary and secondary endpoints	15 (13.9%)
Yes, but partially	59 (54.6%)
No	34 (31.5%)
**Reporting of actual probability values**	
Yes	73 (67.6%)

## Discussion

After a quantitative evaluation of the quality of clinical evidence presented for OMPs at the time of registration for marketing authorization, we found that some studies exhibit methodological flaws i.e. the lack of QoL-related endpoints as outcome, lack of blinding in the study design and the use of surrogate endpoints. To a lesser extent, the lack of dose finding studies is also worrisome from a safety perspective. Additionally, there are important shortcomings in the reporting of those studies, which further complicates the interpretation of the clinical evidence.

### Analysis of the indications

Despite the fact that protocol assistance appears to be positively associated with success of marketing authorization, we found that for just one in four indications, protocol assistance was requested by the sponsor [15]. For only 55% of the indications, a dose-finding study was performed prior to the pivotal study(/ies). This number is on the rise according to Joppi *et al.*[5].

### Analysis of the characteristics of the pivotal studies

Surrogate endpoints were used in a large majority of the studies. Indeed, if a hard endpoint (such as overall survival) is used, it is more difficult to demonstrate an effect given the small sample size and the limited duration of the majority of the studies. Also, fewer patients are required to show a change in a continuous variable (i.e. a surrogate endpoint such as a biomarker) [16]. Additionally, surrogate endpoints can provide guidance for adequate dose selection [17]. The use of these surrogate endpoints seems to have contributed significantly to the rise in numbers of new OMPs [7]. The clinical importance of those surrogate endpoints has however been questioned, as there is not always a strong relationship with clinical meaningful endpoints [5,18]. Still, obtaining marketing authorization based solely on hard endpoints is not always feasible and could jeopardize early access to the market. Therefore, we argue strongly in favour of using at least one hard endpoint in a post-marketing phase IV study. Also, in order to sufficiently capture what is valued by patients, it is recommended to consult with patient(s) (organizations) in an early stage on what endpoints should be considered.

The pivotal studies supporting the marketing authorization of OMPs included between 7 and 976 patients. In a similar study, the small number of patients was also deemed not justifiable for a number of OMPs [5]. Yet, most rare diseases have a prevalence of less than 1 in 10 000 individuals. Given the difficulties to recruit patients for clinical studies, registries of prospectively enrolled patients can be a powerful tool to collect more data. Additionally, those registries can improve our understanding of the natural course of a disease and its relevant biomarkers [19,20].

Registration of clinical studies on EUdraCT is mandatory for all interventional clinical studies in the European Union from 2004 onwards [21]. Similarly, ClinicalTrials.gov offers an overview of publicly and privately supported international clinical studies, but not all studies are required by law to be registered [22]. This implies that not all studies are registered on one or both databases. It is more worrisome that accordance with GCP guidelines was not reported for all studies.

### Analysis of the methodological quality of the pivotal studies

We found that for the majority of the studies the allocation was randomized and/or a control arm was used in the study design. These findings are consistent with another study that reported more use of randomization in the last years [5]. Also, just half of the studies applied some type of blinding. It has been shown that un-blinded studies are more likely to show clinical improvement than blinded studies. Randomized and double blind designs reduce bias [23]. In general, it is assumed that evidence obtained by randomized studies is better than non-randomized. Nonetheless, results from non-randomized studies can still be of use [16]. Bayesian statistical methods have been developed to form prior probability distributions based on non-randomized studies, which can then be combined with randomized evidence [19]. Additionally, well-understood adaptive designs can offer the necessary flexibility and efficiency, if used with due caution [24]. Clearly, these small study populations and/or adaptive study designs can impose a statistical challenge, which is illustrated by the observation that in most studies the statistics are unclear and lacking in detail (i.e. lack of p-values, use of Last Observation Carried Forward to deal with missing data, no correction for baseline imbalances between groups, etc.).

We found that in less than half of the studies, an adequate number of patients, as suggested by a priori power calculations, was included. Performing underpowered studies has been regarded as unethical, because study participants are unable to contribute to improved care for future patients [25]. Also, the appropriateness of the study population is a cause for concern. For example, including patients with minimal symptoms (so called “atypical cases”), while excluding children, females, patients with severe end-organ damage etc. can skew the population and affect the interpretation. Furthermore, the characteristics of the study population should be reflected in the choice of endpoints (and not vice versa). For example, no small children were included in the pivotal study for galsulfase, because the primary endpoint was a 12 minute walking test.

Even though QoL is a highly relevant outcome in the evaluation of chronic and debilitating diseases, only a minority of the studies included a QoL-related endpoint. Additionally, an improvement in QoL was obtained in just ten studies. Disease-specific QoL measures, although preferred because of their responsiveness to changes in the condition caused by treatment, are not available for all rare diseases. Yet, the use of validated generic QoL measures has become widely accepted [26]. Even for children, there are a few generic Qol measures both proxy and/or self-complete available [27]. Higher quality efficacy data could possibly be attained by imposing the use of Quality-of-Life (QoL) related endpoints.

The duration of the study was considered too short, relative to the disease, for approximately a fifth of all studies. Similarly, the duration and the length of follow-up were considered to be too short in other studies [5,8]. In some cases, the duration of a study was sufficient to demonstrate a treatment effect based on the predefined endpoints. But, the validity of some (surrogate) endpoints has been questioned, as some do not translate into clinical benefit nor are in line with the natural course of a disease [28]. For example, the pain endpoint used in a study for agalsidase alfa has been refused by the Food and Drug Administration (FDA) [29]. Also, the pattern of expected prognosis may also change over time as diagnosis improves and background of standard of care improves [30].

### Analysis of the quality of reporting of the pivotal studies in the EPAR

The assessment of the methodology quality also depends on the information available in the EPARs. The conciseness of the information in the EPAR made the analysis of nine pivotal studies impossible. Additionally, we found that some items, such as a description of the patients, the point estimates and measures of variability for all endpoints and the actual probability variables are not reported for all studies. With a view to interpreting the results, these elements, together with the appropriate probability values, are indispensable. Unlike journal articles, EPARs are not subject to space constraints. Therefore lack of information could lead to the false assumption that a study was methodologically deficient [23]. In order to set up more uniform requirements for reporting, a checklist was drawn up, following actions agreed upon by EUnetHTA and EMA, for the EPAR improvement project [31].

This study has several strengths and weaknesses. It provides a quantitative evaluation of the level of clinical evidence presented in all pivotal studies for all OMPs. Additionally, the results were validated by clinical experts. However, the study is limited to the EPAR and/or SD documents and it therefore subject to bias by the quality of reporting in these documents. As such, it does not take into consideration the (possibly relevant) evidence that was generated after obtaining marketing authorization and/or in publications (which may contain more details on for example inclusion criteria and would thus allow for quality control of the included data). For example, a randomized, double-blind, placebo-controlled study was conducted for agalsidase alfa after marketing authorization [32]. Also, we did not categorize our results based on the type of marketing authorization (ie normal, conditional, under exceptional circumstances), but opted to show aggregate data. Finally, we did not include non-OMPs in the analysis; as such it remains unclear whether similar issues apply to non-OMPs.

## Conclusions

In conclusion, the pivotal studies that are the basis for marketing authorization of OMPs are a cause for concern. Considering that these products have a substantial impact on the health care budget, it is clear that a balance must be struck between stimulating marketing authorization and requesting more and higher-quality efficacy data [8]. Requests for better evidence for OMPs can be acceded to through new adaptive study designs [33]. Additionally, the creation of European registries for each OMP, as a post-marketing commitment, could prove to be beneficial in collecting long-term clinical data [34]. A more demanding regulatory process for OMPs is needed to guide evidence-based clinical decision-making.

## Abbreviations

COMPASS: Clinical evidence of Orphan Medicinal Products-an ASSessment tool; OMP: Orphan medicinal product; EU: European Union; QoL: Quality-of-life; EPAR: European Public Assessment Report; FDA: Food and Drug Administration; SD: Scientific Discussion; CHMP: Committee for Human Medicinal Products; EMA: European Medicines Agency.

## Competing interests

EP, DC, JAM and SS declare that they have no competing interests. CEH received reimbursement of expenses or honoraria for lectures on the management of lysosomal storage diseases from Genzyme Corporation, Shire and Protalix. All honoraria are donated to the Gaucher Stichting, a national foundation that supports research in the field of lysosomal storage disorders.

## Authors’ contributions

The work presented was carried out in collaboration between all authors. EP, SS, DC and BA were involved in the initiation of the project and the design of the methods. EP, DC, CEM, JAM and SS actively participated in the data collection. EP analysed the data and interpreted the results. EP, DC and SS discussed the results. EP and SS wrote the draft paper. SS, DC, CEM and JAM revised the draft paper. All authors have read and approved the final manuscript.
